# Ectopic PDX-1 Expression Directly Reprograms Human Keratinocytes along Pancreatic Insulin-Producing Cells Fate

**DOI:** 10.1371/journal.pone.0026298

**Published:** 2011-10-18

**Authors:** Michal Mauda-Havakuk, Naomi Litichever, Ellad Chernichovski, Odelia Nakar, Eyal Winkler, Ram Mazkereth, Arie Orenstein, Eran Bar-Meir, Philippe Ravassard, Irit Meivar-Levy, Sarah Ferber

**Affiliations:** 1 Sheba Regenerative Medicine, Stem Cells and Tissue Engineering Center, Sheba Medical Center, Tel-Hashomer, Israel; 2 Department of Human Genetics and Molecular Medicine, Sackler School of Medicine, Tel-Aviv University, Tel-Aviv, Israel; 3 Department of Plastic and Reconstructive Surgery, Sheba Medical Center, Tel-Hashomer, Israel; 4 Albert Katz Department of Neonatology, Sheba Medical Center, Tel-Hashomer, Israel; 5 Biotechnology and Biotherapy group Centre de Recherche Institut du Cerveau et de la Moelle CNRS UMR7225, INSERM UMRS795, Université Pierre et Marie Curie, Paris, France; Universidade Federal do Rio de Janeiro, Brazil

## Abstract

**Background:**

Cellular differentiation and lineage commitment have previously been considered irreversible processes. However, recent studies have indicated that differentiated adult cells can be reprogrammed to pluripotency and, in some cases, directly into alternate committed lineages. However, although pluripotent cells can be induced in numerous somatic cell sources, it was thought that inducing alternate committed lineages is primarily only possible in cells of developmentally related tissues. Here, we challenge this view and analyze whether direct adult cell reprogramming to alternate committed lineages can cross the boundaries of distinct developmental germ layers.

**Methodology/Principal Findings:**

We ectopically expressed non-integrating pancreatic differentiation factors in ectoderm-derived human keratinocytes to determine whether these factors could directly induce endoderm-derived pancreatic lineage and β-cell-like function. We found that PDX-1 and to a lesser extent other pancreatic transcription factors, could rapidly and specifically activate pancreatic lineage and β-cell-like functional characteristics in ectoderm-derived human keratinocytes. Human keratinocytes transdifferentiated along the β cell lineage produced processed and secreted insulin in response to elevated glucose concentrations. Using irreversible lineage tracing for KRT-5 promoter activity, we present supporting evidence that insulin-positive cells induced by ectopic PDX-1 expression are generated in ectoderm derived keratinocytes.

**Conclusions/Significance:**

These findings constitute the first demonstration of human ectoderm cells to endoderm derived pancreatic cells transdifferentiation. The study represents a proof of concept which suggests that transcription factors induced reprogramming is wider and more general developmental process than initially considered. These results expanded the arsenal of adult cells that can be used as a cell source for generating functional endocrine pancreatic cells. Directly reprogramming somatic cells into alternate desired tissues has important implications in developing patient-specific, regenerative medicine approaches.

## Introduction

Adult cells in mammals were considered terminally differentiated. The differentiation of diverse cell types is achieved by specific transcription factors that reinforce cell-type-specific gene expression patterns; these patterns are stabilized by epigenetic modifications that can be transmitted to daughter cells [1]. However, it was recently established that the fate of adult somatic cells is not rigidly fixed; in fact, adult somatic cells have a substantial amount of developmental plasticity [2]–[4].

The remarkable transformation of adult cells into pluripotency has been interpreted as a reversion from the mature state into a primitive developmental state, which, in many aspects, resembles embryonic stem cells [5], [6]. However, the forced expression of lineage-specific developmental factors in adult cells induced traits of various committed cell types without first inducing pluripotency [7]–[10] The first example of functional adult cell reprogramming to the pancreatic lineage in vivo, was documented over a decade ago [7]. Numerous research groups have been successful in activating the pancreatic lineage in liver cells from *Xenopus laevis* [11], rodents [7], [12]–[17], and humans [18]–[21], in vivo and in vitro, respectively. It was suggested that the ectopic expression of a dominant pancreatic transcription factor, PDX-1, played a critical role in the reprogramming process [12], [19], [22]–[24]. Furthermore, increased efficiency of the reprogramming process was achieved by co-expressing PDX-1 with one or several other pancreatic transcription factors, including MAFA, NGN3, and/or NEUROD1 [16], [17], [25], [26].

The success in activating the endocrine pancreatic lineage in liver or exocrine pancreas [27], [28] has been attributed to the close developmental relationship between the transconverting tissues; that is, they all originated from the primitive foregut endoderm [29]–[31]. However, the current understanding of stem cell biology and induced pluripotency favors the notion that every adult tissue may have the potential to undergo reprogramming to a pluripotent state in response to defined developmental factors [32]. The fact that adult cells can be reprogrammed into pluripotency suggests that cellular integrity is preserved through adulthood by epigenetic barriers that can be reversed with developmental stimuli. The general notion that adult cells possess this plasticity suggests that it may be possible to reprogram adult somatic cells directly to committed lineages, including the pancreatic lineage.

This study aimed to determine whether the ability to reprogram adult cells directly into the endocrine pancreatic lineage and confer pancreatic function could be applied to a broader spectrum of cell types than previously suggested. We hypothesized that reprogramming may be possible between developmentally unrelated tissues. To test this, we focused on reprogramming ectoderm-derived human skin keratinocytes into the endoderm-derived pancreatic lineage. We chose ectoderm-to-endoderm lineage reprogramming, because cells from distinct developmental germ layers have pronounced developmental disparity. We tested pancreatic transcription factors for the capacity to directly activate the pancreatic lineage and β-cell-like function without first inducing pluripotency in human keratinocytes.

## Methods

### Ethics statement

Human skin tissues were obtained from neonatal foreskins (8 days old) and from split thickness skin grafts (1–4 cm^2^), taken from 9–70 years old donors, with approval from the Committee on Clinical Investigations (institutional review board, Sheba MDC). All donors provided written informed consent for the collection of all samples and subsequent analysis.

### Cell culture

3T3 cells were grown at 37°C in humidified incubator with 5% CO_2_, in DMEM (high, 4.5 g/l glucose), 10% FCS (Gibco), 2 mM l-glutamine, 100 U/ml penicillin, 100 µg/ml streptomycin and 0.25 µg/ml Amphotericin B (all from Biological Industries, Beit Haemek, Israel). To block 3T3 cell proliferation, cells at 70% confluency were incubated with Mitomycin-c (4 µg/ml, Sigma) for 90 min. The cells were subsequently washed by PBS and cultured in DMEM for additional 24 h before used as a feeder layer for the human keratinocytes.

### Primary culture of human keratinocytes

Keratinocytes isolation and culture were performed as previously described [33]–[35]. Skin tissues were cut to 2–3 cm^2^ pieces, washed twice in PBS and incubated overnight at 4°C in 2 U/ml Dispase (Gibco). The epidermis was separated and incubated in trypsin at 37°C for 10 min, cells were collected by centrifugation and seeded on the mitomycin c treated 3T3 cells at the density of 10^7^ cells/100 mm plate. The culture media used was Green medium (Green et al [33]–[35]), composed of F-12 Nutrient mixture with DMEM High glucose (1∶3), 2 mM l-glutamine, 100 U/ml penicillin, 100 µg/ml streptomycin, 0.25 µg/ml Amphotericin B, 0.05 mg/ml Gentamycin Sulphate (Biological Industries, Beit Haemek, Israel), 10% FCS (Gibco), 10 ng/ml Epidermal Growth Factor, 10^−10^ M Cholera Toxin, 0.4 µg/ml Hydrocortisone, 2 nM T3, 5 µg/ml Transferrin, 5 µg/ml Insulin, 0.18 mM Adenine (all from Sigma). Within a week or two the keratinocytes grow and filled the plate, displacing the 3T3 cells. Subsequently, the cells were split once a week as the cells reached confluence but before the cells started to undergo spontaneous stratification. Cells detachment was performed by trypsin, and the cells were seeded without the feeder layer at 10^6^ cells/100 mm plate, for up to 6 passages.

### Viral infection

For adenovirus infection, keratinocytes at passage 2–4 were plated at 3×10^6^ cells/100 mm plates and infected with the relevant recombinant adenovirus on the next day at 10–1000 moi (multiplicity of infection). The following adenoviruses were used in this study: *Ad-CMV-PDX-1* (replication-deficient recombinant adenovirus that encodes rat PDX-1 cDNA under the control of the cytomegalovirus promoter,[36]); *Ad-CMV-GFP* (Clontech, Mountain View, CA); *Ad-CMV-β-gal* (a gift from C.B. Newgard, Duke, NC); *Ad-RIP-GFP* [18]; *Ad-RIP-luciferase* [36].

### Luciferase assay

Keratinocytes were co-infected with *Ad-RIP- luciferase* (200moi) and *Ad-CMV-PDX-1* or *Ad-CMV-β-gal* as control (both at 1000moi). Seven days later, lucifease activity was measured using Lucifersase assay System (Promega) by LKB 1250 Luminometer (LKB, Finland). The results were normalized to total cellular protein measured by the Bio-Rad Protein Assay kit (Bio-Rad).

### Letniviruses production and infection

The two component lentivirus system described by Russ et. al. [37], was used to irreversibly label keratinocytes for KRT5 expression manifested by its promoter activity. Human keratinocytes at passage 2 were co-infected with the Reporter vector CMV-loxP-DsRed2-loxP-eGFP (R/G) and a vector which carries the expression of Cre-recombinase under the control of the KRT5 promoter (a gift from P.W. Zoltick [38]). R/G treatment resulted in DsRed2 but not eGFP expression. The KRT5 promoter activates Cre-recombinase expression only in KRT5 positive cells which cleaves the “floxed” DsRed2, allowing the expression of eGFP under the CMV promoter. Keratinocytes which are positive to KRT5 and co-express both lentiviruses are expected to activate eGFP and lose the original DsRed2 labeling [37]. Ten days subsequent to lentivirus infection, keratinocytes were treated to ectopically express pancreatic transcription factors, using recombinant adenoviruses.

### RNA isolation, RT and RT-PCR reactions

Total RNA was isolated, cDNA was prepared and amplified as described previously [12], [18]. Quantitative real-time RT-PCR was performed using ABI StepOnePlus sequence Detection system (*Applied Biosystems*, CA, USA) as described previously [18], [20], [39]. The primer sets used in this study are listed in Methods S1.

### Immunoflorescence

Keratinocytes were seeded on cover-slips and infected with the indicated adenovirus on the next day. Seven days later cells were fixed and stained as previously described [18], [20], [39]. The antibodies used in this study were: anti mouse KRT5 (1∶100), anti human P63 (1∶100) anti human KRT8/18 (1∶200), anti human Ki67 (1∶150)(all from Abcam, Cambridge UK), anti human C-peptide (1∶200 Biodesign, Maine, USA), anti human glucagon (1∶200, Dako, Glostrup, Denmark), anti mouse NKX6.1 (1∶6000 a generous gift from C.B. Newgard, Duke University), anti mouse NEUROD1 (1∶00, Santa Cruz Biotechnology, Santa Cruz, CA, USA), anti mouse NGN3 (1∶50) anti chick NKX2.2 (1∶50) both from Developmental Studies Hybridoma Bank, Iowa, USA), anti-CRE (1∶1,000; Novagen), anti-rabbit PDX-1, anti- goat PDX-1 (both 1∶10000 a generous gift from C.V. E. Wright). Anti-Secondary anti-rabbit IgG Cyanine (cy2) conjugated antibody, anti-rabbit IgG indocarbocyanine (cy3) conjugated antibody, anti-goat IgG Cyanine (cy2) conjugated antibody, anti-goat IgG indocarbocyanine (cy3) conjugated antibody, anti-mouse IgG Cyanine (cy2) conjugated antibody, anti-mouse IgG Indodicarbocyanine (cy5) conjugated antibody, anti-mouse IgG Aminomethylcoumarin (AMCA) conjugated antibody and anti-mouse IgG indocarbocyanine (cy3) conjugated antibody (all 1∶250 from *Jackson ImmunoResearch*, PA). Finally, the cells were stained with 4′, 6-diamidino-2-phenyl-indole (DAPI, *Sigma*). The slides were analyzed using a fluorescent microscope (Provis, Olympus), or Laser Scanning Confocal microscopy (*Bio-Rad*, LSM-1024).

### C-peptide secretion

C-peptide secretion was measured by static incubations of keratinocytes cells, 7 days after the initial exposure of the cells to the adenovirus treatment as described [18], [39]. The glucose regulated insulin and c-peptide secretion were measured at 2 and 25 mM glucose or 2 Deoxy-D-glucose, at similar concentrations. C-peptide secretion was detected by radioimmunoassay using human C-peptide radioimmunoassay kit (Linco Research, St. Charles, MO; <4% cross-reactivity to human proinsulin/insulin). The secretion was normalized to the total cellular protein measured by a Bio-Rad protein assay kit.

### Statistical Analyses

Statistical analyses were performed with a 2-sample Student *t* test assuming unequal variances

## Results

### Characterization of primary human keratinocyte cultures

**Figure 1:**
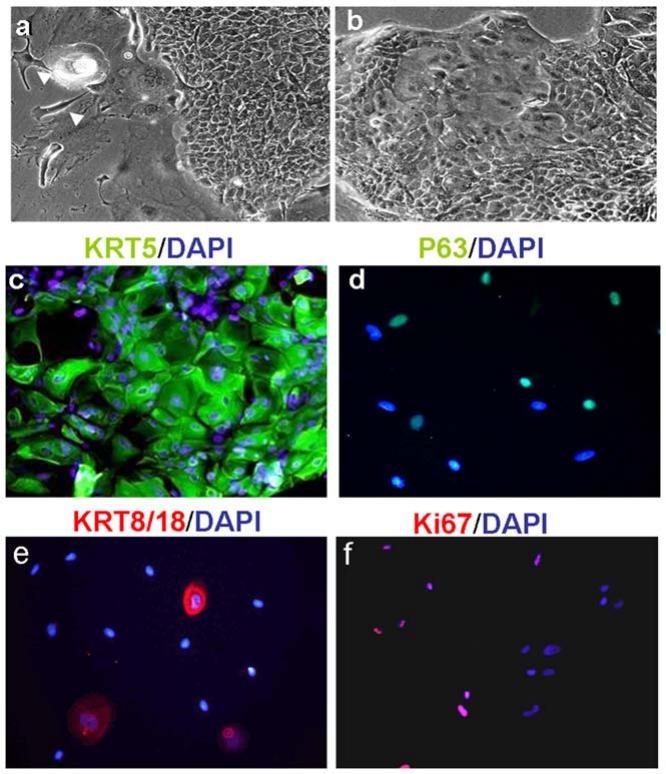
Characterization of primary culture of human keratinocytes *in-vitro*. Human keratinocytes proliferated efficiently for up to 5–7 passages in culture. (a, b) Representative phase contrast images show the morphology of keratinocytes at passages (a) 0, and (b) 3. Arrowheads (a) indicate cells of the 3T3 fibroblast feeder layer in passage 0; original magnification ×10. (c–f) Keratinocytes (passage 3) were stained for keratinocyte markers: (c) KRT5, (d) P63, (e) KRT8/18, and (f) for the proliferation marker, Ki67. Nuclei were stained with DAPI (blue); original magnifications, ×20.

Monolayer cultures of human keratinocytes derived from both neonates and adults were allowed to proliferate for up to 5–6 passages (Fig. 1, 25% Ki67 positive cells). Over 85% of the cells expressed the keratin-5 protein (KRT5; Fig. 1c, d), but most were negative for the KRT1/10 protein (immunofluorescence data not shown). Approximately 10–15% of the cells expressed the KRT8/18 cytokeratins, which are specifically expressed in simple epithelium. Thus, these primary cells were in an early stage of epidermal differentiation in culture (Fig. 1e) [40]. About 60% of the cells in culture were positively stained for P63 nuclear factor, which is a homolog to the tumor suppressor P53 and plays a critical role in the development of stratified epithelia, like the epidermis [41]. These keratinocytes were efficiently infected (70–90%) *in vitro* with the adenoviral vector, *Ad-CMV-GFP* (1000 moi, data not presented).

### Expression of pancreatic transcription factors activate pancreatic hormone gene expression in adult and neonate human keratinocytes

**Figure 2:**
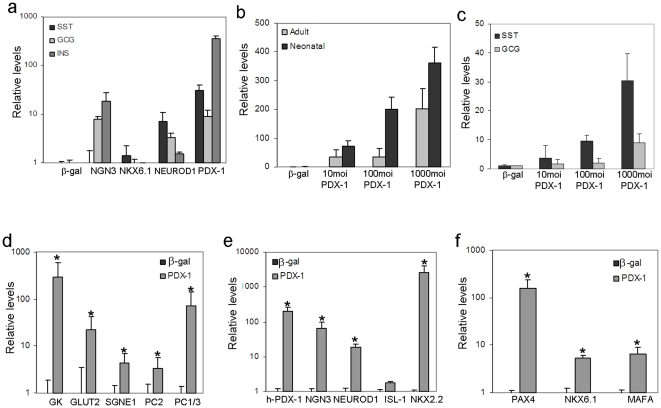
Ectopic PDX-1 expression promoted pancreatic differentiation in human keratinocytes. (a) Human keratinocytes isolated from neonatal foreskin were analyzed by quantitative RT-PCR to determine insulin (*INS*), glucagon (*GCG*), and somatostatin (*SST*) gene transcription levels induced by adenoviral infections with transcription factor genes *PDX-1*, *NGN3*, *NKX6.1*, or *NEUROD1*. The control virus carried only the *β-gal* gene sequence. All vectors were infected at 1000 moi and cells were analyzed 7 days post infection. (b) Human keratinocytes isolated from neonatal foreskin or adult skin grafts, (9–70 year-old donors) were infected with increasing concentrations of *PDX-1* (10–1000 moi) or control (*β-gal*) adenovirus constructs to determine *INS* gene transcription. (c) Adult keratinocytes were infected with increasing concentrations of *PDX-1* (10–1000 moi) or control (*β-gal*) adenovirus constructs to determine *GCG* and *SST* gene transcription levels. (d, e, f) Keratinocytes from neonatal foreskin were infected with *PDX-1* or control (*β-gal*) adenovirus constructs (both at 1000 moi) to determine transcription levels of genes encoding (d) pancreatic-specific proteins, (e) pancreatic specific transcription factors, and (f) β-cell specific transcription factors. The results are normalized to *β-actin* gene transcription within the same cDNA samples. The mean ±standard deviations are expressed as the signal strength relative to cells treated with the control virus. All results represent n≥8 samples tested in three independent experiments *P<0.01.

Transcription factors that are instrumental in driving pancreatic organogenesis in the embryo demonstrated a similar role in reprogramming adult endoderm-derived cells to follow the pancreatic lineage. Several transcription factors, including PDX-1 [12], [19], [22]–[24], NEUROD1 [42], or NGN3 [26] have exhibited individual roles in liver cell transdifferentiation to the pancreatic lineage. To determine whether these factors could induce a similar developmental process in cells of developmentally unrelated tissues, we tested them individually for activating pancreatic hormone gene expression in human keratinocytes. Ectopic expression of pancreatic transcription factors was mediated by viral infection with non-integrating, recombinant adenoviruses, *Ad-CMV-PDX-1*, *Ad-CMV-NGN3*, *Ad-CMV-NEUROD1*, *Ad-CMV-MAFA*, or *Ad-CMV-NKX6.1* (all at 1000 moi). Ectopic NKX6.1 expression could not activate pancreatic hormone gene transcription in keratinocytes, consistent with its lack of individual effect on liver cells [21]. Ectopic NGN3 expression activated insulin and glucagon, but not somatostatin transcription. Both PDX-1 and NEUROD1 ectopic expression activated the transcription of all three pancreatic hormones. However, PDX-1 induced a higher level of hormones gene expression than NEUROD1(Fig. 2a). Therefore, we focused on the individual ability of PDX-1 to drive the developmental fate of human keratinocytes to the pancreatic lineage.

### PDX-1 activates transcription of numerous pancreatic markers in keratinocytes

**Figure 3:**
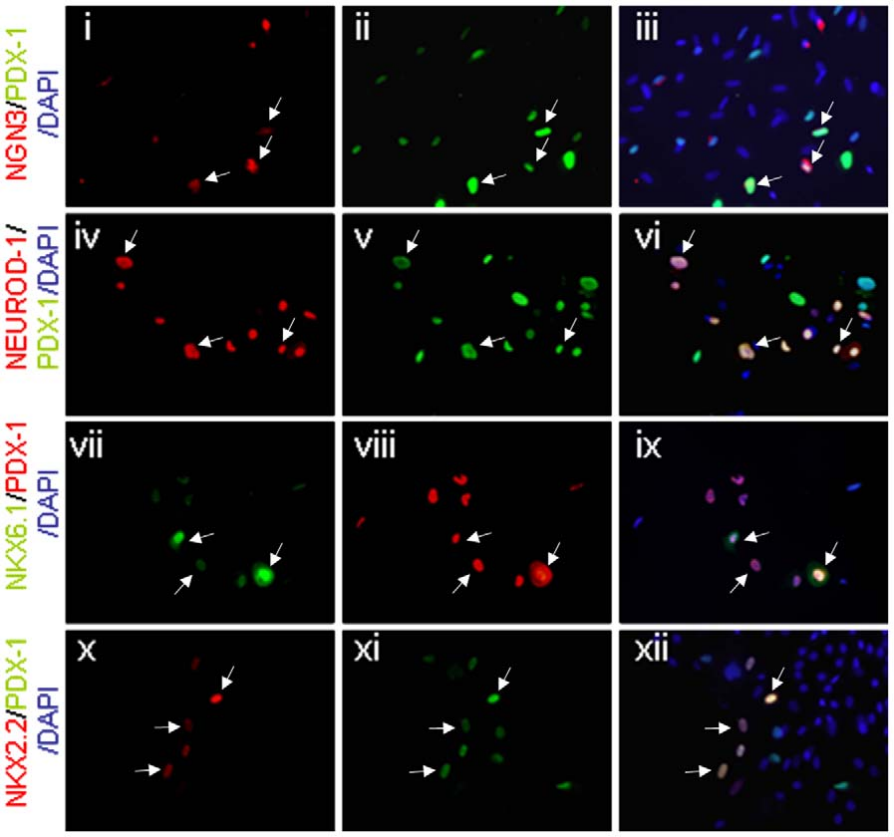
Nuclear localization of PDX-1 induced pancreatic transcription factor expression in human keratinocytes. Human keratinocytes isolated from neonatal foreskin were analyzed 7 days subsequent to Ad-PDX-1 infection (ectopic PDX-1). Double Immunofluorescence analyses showed co-localization of (i, ii, iii) NGN3 (red) and ectopic PDX-1 (green); (iv, v, vi) NEUROD1 (red) and ectopic PDX-1 (green); (vii, vii, ix) NKX6.1 (green) and ectopic PDX-1 (red); and (x, xi, xii) NKX2.2 (red) and ectopic PDX-1 (green). In panels iii, vi, ix, and xii, the images were superimposed to visualize co-localization (merged colors). Nuclei were stained with DAPI (blue). Original magnification ×20; arrows indicate double-stained cells.

**Figure 4:**
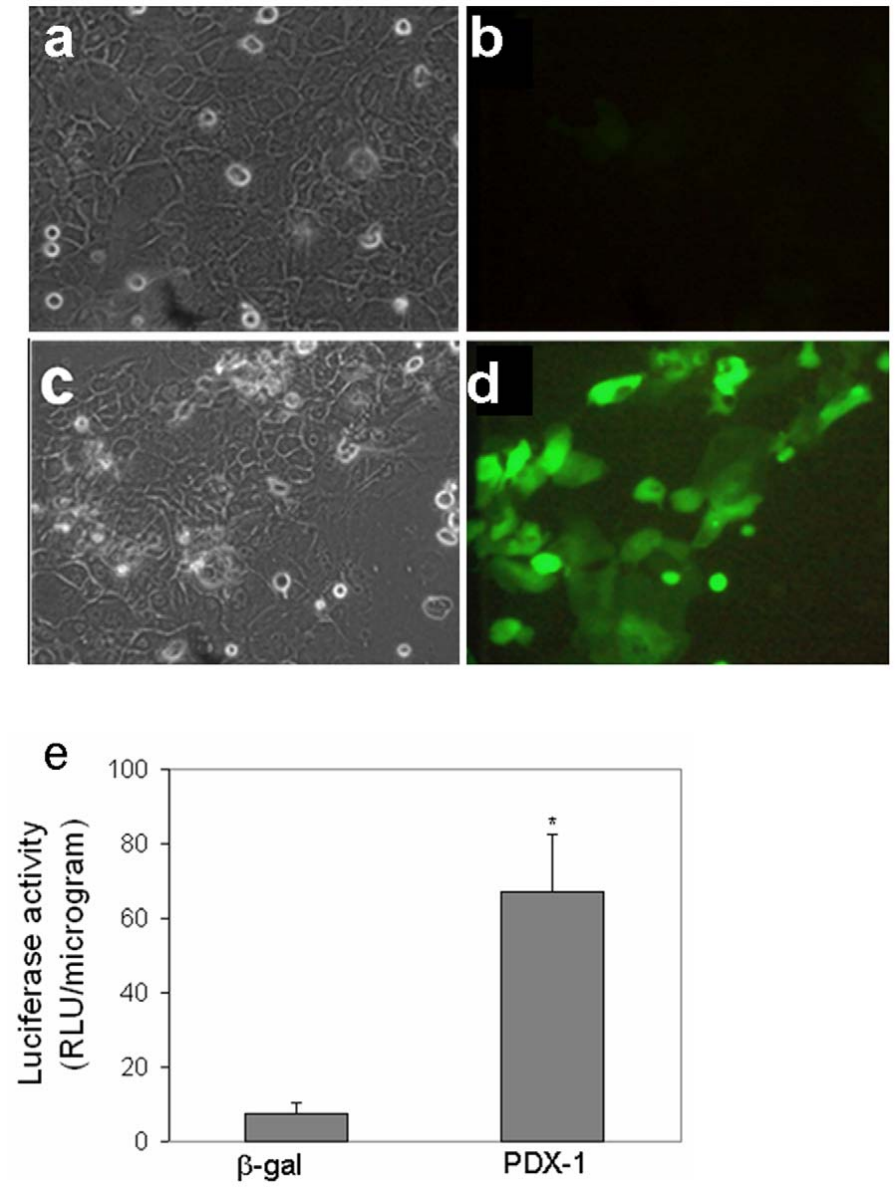
Ectopic PDX-1 expression activated the insulin promoter in human keratinocytes *in-vitro*. Human keratinocytes isolated from neonatal foreskin were co-infected with either the control virus *Ad-β-gal* or *Ad-PDX-1* (both at 1000 moi) and a reporter gene of either (a–d) *Ad-RIP-GFP* (1000 moi,) or (e) *Ad-RIP-Luciferase* (100 moi,), for 7 days. (a, c) Representative phase contrast micrographs show cell morphology. (b, d) Green fluorescent protein imaging of the same fields (a&b; c&d) presented in the phase contrast. (b) control cells did not express GFP; and (d) PDX-1 stimulated GFP expression in numerous cells. (e) Luciferase activity of the infected keratinocytes. The mean ±standard deviation is expressed in relative luciferase units; data represents n≥8 samples in three independent experiments.

Expression of PDX-1 in human keratinocytes caused the transcription of numerous genes characteristic of the pancreatic lineage and β-like-cells (Fig. 2), without affecting cell morphology (Fig. 3a, c). This effect of PDX-1, but not control virus, was dose dependent in both neonatal and mature human keratinocytes (Fig. 2b, c). Due to their ready availability and higher responsiveness, we performed further analyses in neonatal-derived human keratinocytes. PDX-1 expression activated the expression of genes involved in β-cell glucose sensing (GLUT-2, GK), prohormone processing (PC1/3 and PC2), and secretion granule assembly (SGNE1; Fig. 2d). These genes, characteristic of endocrine cells, were silent in untreated or control treated keratinocytes. The considerable pancreatic repertoire induced by PDX-1 could partly be attributed to its marked activation of numerous pancreatic-specific transcription factor genes (Fig. 2e, f), including *NGN3*, *ISL1*, *NEUROD1*, *NKX2.2*, *NKX6.1*, *MAFA*, *PAX4*, and the endogenous human *PDX-1*. Importantly, many of the treated human keratinocytes exhibited co-localization of PDX-1 and the induced transcription factors in the nucleus (Fig. 4); 51% of PDX-1 positive cells were positive to NGN3; 41% were positive to NKX2.2; 45% were positive to NKX6.1 and 46% were positive to NEUROD1. Taken together these data suggest that PDX-1 induced a wide developmental alteration when ectopically expressed in human keratinocytes.

### The efficiency of PDX-1 induced keratinocytes reprogramming along the endocrine pancreatic lineage

To examine the efficiency of reprogramming skin keratinocytes to become insulin-producing cells, we determined the percentage of PDX-1 treated keratinocytes that were capable of activating an ectopically expressed insulin promoter.

Human keratinocytes were co-treated with either *Ad-CMV-PDX-1* or *Ad-CMV-β-gal* control recombinant adenoviruses (1000 moi) and *Ad-RIP-GFP*, which carries the *GFP* reporter gene sequence under the control of the rat insulin promoter 1 [18]. Twenty five percent of cells were capable of activating the ectopic pancreatic insulin promoter upon PDX-1 infection; no signal was detected with control adenovirus infection (Fig. 3a–d). The PDX-1 dependent activation of the insulin promoter was further confirmed in a Luciferase reporter (*Ad-RIP-Luciferase*) assay. This showed that the insulin promoter was significantly stimulated by PDX-1 (Fig. 3e).

**Figure 5:**
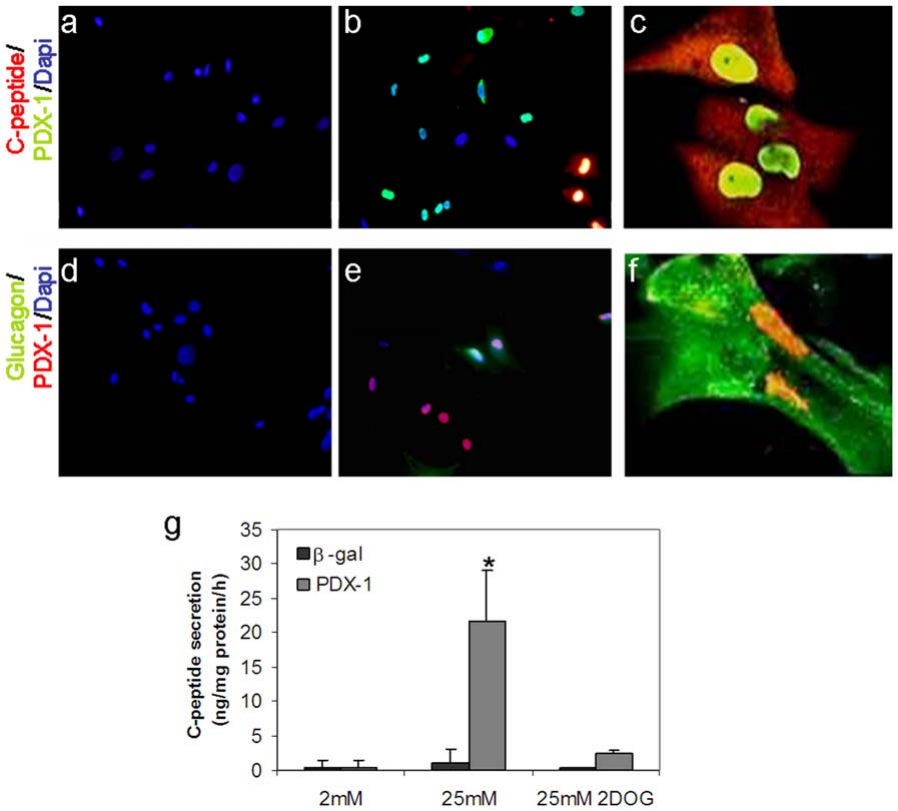
Ectopic PDX-1 expression induced insulin and glucagon expression and promoted glucose-regulated C-peptide secretion. Double immunofluorescence of human keratinocytes isolated from neonatal foreskin treated with (a, d) control *Ad-β-gal* or (b–c, e–f) *Ad-PDX-1* adenovirus for 7 days. Immunofluorescence analyses showed co-localized expression of (a–c) PDX-1(green) and insulin (red) and (d–f) PDX-1(red) and glucagon (green). The nuclei were stained with DAPI (blue). (a–b, d–e). Original magnification ×20 (c, f); original magnification (confocal microscope) ×90. (g) Human keratinocytes were infected with *Ad-β-gal* or *Ad-PDX-1*, both at 1000 moi, and analyzed for secretion of processed insulin into the culture medium upon exposure to 2 and 25 mM glucose or 25 mM 2DOG. C-peptide in the medium was measured with a specific RIA kit (Linco human c-peptide no crossreactivity with pro-insulin). Data represents n≥7 samples in four independent experiments.

Ectopic insulin promoter activation provides an essential clue on the reprogramming efficiency; however, it does not reflect changes in the epigenetic status of the host cells, as the adenovirus reporter gene (*Ad-RIP-GFP or Ad-RIP- Luciferase*) does not integrate into the host genome. A more stringent indication for reprogramming toward β-cell lineage can be obtained by analyzing endogenous insulin gene expression (Fig. 2b) and hormone production (Fig. 5). Indeed, 12.61±8% of PDX-1 positive cells stained positive for the endogenous insulin (C-peptide, detected with the anti-human C-peptide antibody; Biodesign; Fig. 5b & c). Ectopic PDX-1 expression also activated glucagon production in human keratinocytes (Fig. 5e & f). Co-localization of PDX-1 in glucagon producing cells, may suggest these cells immature characteristics. Taken together these data demonstrate the capacity of PDX-1 to activate the pancreatic lineage in human keratinocytes at the molecular and cellular levels. Although ectopic expression of PDX-1 was detected in 70–90% of all cells in culture, the activation of the pancreatic phenotype occurred only in scattered keratinocytes, which as in liver may be prone to transdifferentiation [18], [20], [22], [39]. The nature or the characteristics of the predisposed cells is not yet understood.

### PDX-1 induced β-cell-like function in human skin keratinocytes

The significant activation of numerous markers and transcription factors characteristic of the endocrine pancreas together with the generation of pancreatic hormones producing cells, motivated us to determine whether PDX-1 also activated β-cell-like function in the ectoderm-derived human keratinocytes.

The most critical demonstration of β-cell-like function is the capacity to produce insulin, sense glucose, and accordingly, secrete the processed hormone [43]. We incubated PDX-1 treated keratinocytes with low (2 mM) or high (25 mM) glucose concentrations and analyzed C-peptide secretion into the media as an indicator of processed insulin (human C-peptide RIA (Linco, <4% cross-reactivity to human proinsulin or insulin)). We found that PDX-1 treated keratinocytes released the processed hormone only upon exposure to high concentrations of glucose (Fig. 5g). A non-metabolizable glucose analog, 2-deoxy-glucose (2-DOG), served both as an indicator of osmolarity and a functional control. We found that a similar concentration of 2-DOG (25 mM) did not trigger C-peptide secretion (Fig. 5g). This suggested that, like pancreatic β-cells, the reprogrammed keratinocytes required glucose metabolism to trigger insulin secretion; thus, glucose metabolism was coupled to the hormone secretion process.

### Reprogramming specificity: PDX-1 activates the expression of early endodermal markers but not that of other adult endodermal tissues, in human keratinocytes

**Figure 6:**
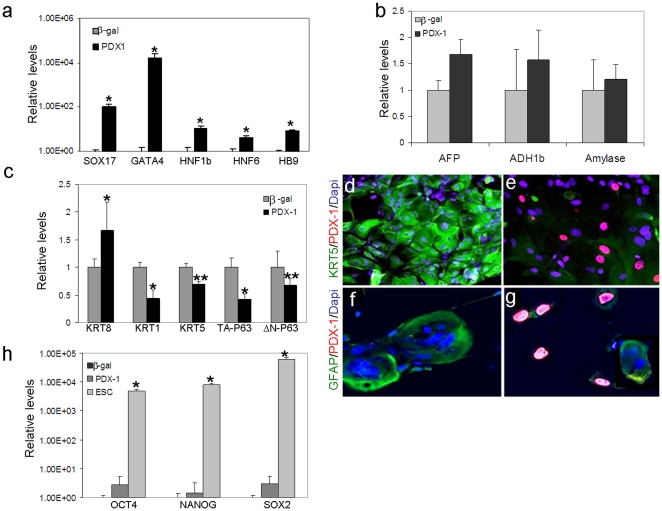
Ectopic PDX-1 activated early endoderm but not hepatic or pancreatic acinar markers, while repressing the expression of keratinocyte-specific markers. Human keratinocytes isolated from neonatal foreskin were infected with *Ad-β-gal* or *Ad-PDX-1* (both at 1000 moi) for 7 days. Quantitative real time RT-PCR analyses indicated the relative transcription levels of (a) early endoderm-specific markers, (b) hepatic and pancreatic acinar markers, (c) keratinocyte markers or (h) embryonic stem cell markers. The results were normalized to the level of *β-actin* gene transcription within the same cDNA sample. The mean ±standard deviations of *Ad-PDX-1* treated cells are expressed relative to the mean levels observed in *Ad-β-gal* treated cells. n≥6 from 3 different cultures. *P<0.01, **P<0.05 (h) cDNA extracted from human embryonic cells was used as positive control. (d–g) Cells treated with either (d, f) *Ad-β-gal* or (e, g) *Ad-PDX-1* were fixed and co-stained for (d, e) KRT5 (green) and PDX-1 (red) or for (f, g) GFAP (green) and PDX-1 (red). The nuclei were stained with DAPI; magnification: d–g ×30.

During pancreas development in the embryo, early endodermal markers expression exceeds that of PDX-1. Surprisingly, in ectoderm-derived cells, PDX-1 activated the expression of numerous early endodermal markers (Fig. 6a). The expression of endodermal markers SOX17 and GATA4 was evident within 48 h while insulin expression was maximally detected 5–7 days after PDX-1 treatment (data not presented).

Despite the activation of the general endodermal markers, neither pancreatic exocrine genes (Amylase), nor hepatic markers (ADH1b and AFP; Fig. 6b) expression was induced in human keratinocytes, suggesting the specificity of the PDX-1 induced reprogramming process.

### PDX-1 inhibits the expression of keratinocytes markers but does not activate embryonic cell markers, in human keratinocytes

Hepatic dedifferentiation has been suggested essential for the activation of the alternate pancreatic repertoire [39]. Moreover, PDX-1 has been suggested to play a dual role in liver to pancreas reprogramming by both repressing the host repertoire of expressed genes, while activating the alternate pancreatic repertoire. To analyze whether PDX-1 plays a similar role in human keratinocytes, we analyzed its effect on typical keratinocytes markers expression. Ad-PDX-1 but not control virus, repressed KRT5, KRT1, KRT10 (Fig. 6c–e and data not presented), and GFAP expression (Fig. 6f&g). Along with the repression of keratinocytes markers, PDX-1 repressed both p63 isoformes expression (TAp63 and ΔNp63; Fig. 6c). These isoforms endow the keratinocytes' transcription factor, p63, a dual role in initiating the epithelial stratification during development and maintaining proliferate potential of basal keratinocytes in mature epidermis [41].

On the other hand, PDX-1 treatment slightly increased the transcription of KRT-8/18, cytokeratin filament proteins that are typically expressed in unstratified basal epithelial cells in skin development [44]. Since KRT-8/18 is also expressed in pancreatic epithelial cells, increases in KRT-8/18 expression could reflect either a developmental regression of KRT5 positive keratinocytes to an earlier developmental stage or a developmental shift of KRT5 positive cells toward the endocrine pancreatic lineage. The fact that PDX-1 repressed the expression of keratinocyte-specific markers and transcription factor, like KRT1, KRT10 and p63 which mark and control keratinocyte stratification, suggested that PDX-1 may perturb normal epidermal differentiation (Fig. 6c). Importantly, PDX-1 induced keratinocytes markers repression was not associated with increased cell proliferation (unaltered Ki67 staining) or with increase in the pluripotent cells markers such as NANOG, OCT-4 and SOX2 expression (Fig. 6h). Taken together these data suggest that PDX-1 induced keratinocytes reprogramming along the pancreatic lineage is associated with restricted developmental regression but not with induced pluripotency.

### Lineage tracing: PDX-1 activates the pancreatic lineage in keratinocytes

Co-localization of insulin (C-peptide) and keratinocyte markers, may suggest that insulin producing cells are indeed generated in keratinocytes upon ectopic PDX-1expression. However, because PDX-1 repressed keratinocyte marker expression (Fig. 6 c–g), we irreversibly tagged keratinocytes for KRT5 expression prior to the ectopic PDX-1 treatment. We used a lentivirus-based dual expression system for tracing cell fate *in vitro* [37]. This genetic manipulation allowed continuous tracking of keratinocytes' fate after the endogenous KRT5 expression vanished. Lentiviral infection of the keratinocytes was performed at passage 2. The reporter virus alone infected 47±10.4% of cells (averaged over 3 cultures). Double lentivirus infections were successful in 22.8±10.4% of cells, based on measurements of the constitutively active CMV-CRE virus (Fig. S1). Expression of eGFP upon DsRed2/eGFP reporter and KRT-5 Cre-recombinase treatment was detected in 20.5±7.4% of cells (Fig. S1). The specificity of KRT5 lineage tracing was exemplified by co staining for the Cre-recombinase protein which was restricted only to KRT5 positive cells (data not presented). Thus, the KRT5 promoter-CRE lentiviral system specifically and irreversibly tagged KRT5-positive cells.

**Figure 7:**
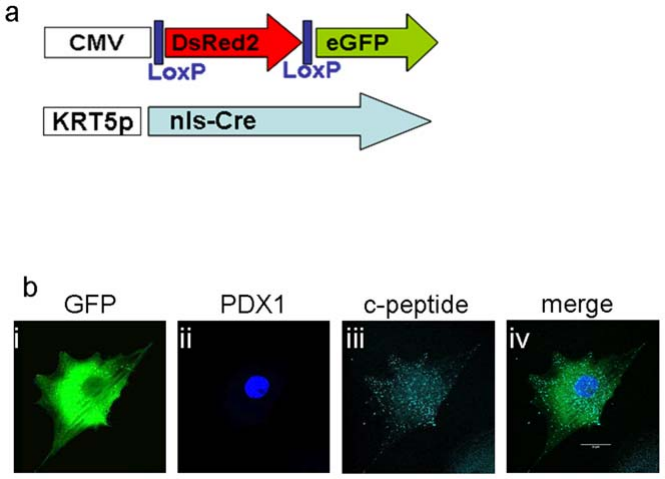
C-peptide positive cells arose from keratinocytes (KRT5 positive cells). Lineage tracing analyses were performed with dual Cre-Lox lentiviral infections. (a) Schematic representation of the Lox (upper) and Cre (lower) lentiviral vectors. Only cells that expressed KRT5 could activate Cre expression; then, Cre would excise the DsRed2 gene between the Lox sites and remove a stop codon preceding the eGFP gene, which is subsequently controlled by the CMV promoter. (b) Ten days after the dual lentiviral infection, cells were treated with *Ad-PDX-1* for 7 days. Fixed cells were stained with three immunofluorescent antibodies and examined with a confocal microscope to detect keratinocytes that processed insulin. (i, ii, iii) GFP (green) identifies the cell as a keratinocyte, PDX-1(blue) shows successful ectopic protein expression, and c-peptide (turquoise) shows processed insulin production. (iv) Superimposed images show that all three proteins are expressed within the same cell. Original magnification ×90.

Keratinocytes irreversibly labeled with KRT5 expression were treated with *Ad-CMV-PDX-1* and immunostained for co-expression of eGFP and C-peptide (Fig. 7b). Indeed, C-peptide positive cells generated upon PDX-1 treatment, also expressed eGFP, suggesting that insulin producing cells can be directly generated in keratinocytes that were positive for KRT5 expression prior to PDX-1 induced reprogramming. The relatively low efficiency of lenti-virus infection (20.5±7.4% of KRT^+^ cells), cannot rule out the possibility that additional cells in the culture could give rise to insulin producing cells. Nonetheless, C-peptide and eGFP co-expression, unequivocally suggest that PDX-1 was capable of activating insulin production in ectoderm-derived human keratinocytes.

## Discussion

The present study demonstrated for the first time the capacity to directly activate the endocrine pancreatic lineage and function in ectoderm derived human keratinocytes. Our data suggested that transcription factors that control pancreas organogenesis such as PDX-1 could activate the pancreatic lineage and function in adult cells of developmentally unrelated tissue in a process which crosses the boundaries of distinct developmental germ layers. This transdifferentiation process is associated with host cells dedifferentiation but not with increased cells proliferation or induction of embryonic markers expression.

PDX-1 treated keratinocytes acquired ‘endocrine-like’ characteristics associated with specific activation of proinsulin processing enzymes, granule assembly markers, and a glucose sensing ability associated with GLUT-2 and Glucokinase (Hexokinase-4) gene expression. None of these properties were detected in control treated keratinocytes (Fig. 2d). The reprogrammed keratinocytes acquired β-cell like functions, including insulin production, processing, and secretion in response to elevated glucose concentration (Fig. 5g). Moreover, like in pancreatic β-cells and in reprogrammed liver cells [18], [45], insulin secretion could only be stimulated by glucose, and similar concentrations of the non-metabolizable glucose analog, 2-DOG had no effect on insulin secretion from reprogrammed human keratinocytes. This indirectly suggested functional coupling between insulin storage compartments and the glucose-sensing apparatus in PDX-1 treated keratinocytes (Fig. 5g).

The reprogramming of human keratinocytes to produce insulin occurred in a relatively short period of time and was relatively efficient, when compared to reprogramming into pluripotency. PDX-1 activated insulin production in 12.61±8% of cells, 5 to 7 days subsequent to Ad-PDX-1 administration. In contrast, reprogramming adult cells into pluripotent cells (iPS) typically takes >20 days [6], [46] with an efficiency of 0.05–0.1%; or 3.3% in clonal cells [5], [6].

Reprogramming ectoderm-derived cells to become endocrine pancreatic-like cells occurred without integration of the developmental factor into the host genome [47]. Ectopic rat PDX-1 delivered by recombinant adenoviruses, activated endogenous human PDX-1 and numerous additional pancreatic transcription factors that are typically silent in this ectoderm derived tissue, and which may have collectively activated the downstream α and β cell specific, adult, functional markers (Figs. 2e–f & 3). The activation of endogenous PDX-1 may suggest that despite the episomal nature of the ectopic PDX-1 gene, the induced reprogramming process may be irreversible, although this should be further directly analyzed. Reprogramming induction in the absence of integration of foreign genetic information has important safety advantage [48]–[51].

Our data suggested that PDX-1 plays a dual role in reprogramming keratinocytes to become β-like-cells, as it both repressed host repertoire of gene expression, while activating the pancreatic lineage protein expression in keratinocytes (Fig. 5 & 6c–g). Since it seems unlikely that *PDX-1* could directly repress expression of multiple genes in the keratinocytes through a direct effect, it could potentially repress a keratinocytes specific transcription factor, which may in turn affect multiple keratinocytes markers expression. Indeed, a similar dual role was described in PDX-1 induced reprogramming of liver along the pancreatic lineage [39]. Hepatic dedifferentiation was obligatory for the PDX-1 induced activation of the pancreatic lineage in liver, and was associated with PDX-1 induced CEBPβ repression [39]. When ectopically expressed in keratinocytes, PDX-1 repressed p63 expression which was suggested essential for epithelial stratification and normal skin development [41]. However, the direct association between keratinocytes dedifferentiation and p63 repression should be further directly analyzed. PDX-1 induced keratinocytes dedifferentiation was not associated by increased cells proliferation or by the activation of early embryonic stem cells markers such as OCT-4, NANOG and SOX-2 (Fig. 6h). This may suggest that restricted developmental regression rather than complete induction of a pluripotent state is sufficient for the activation of the alternate pancreatic repertoire, by ectopic expression of transcription factors of committed cell lineages.

PDX-1 activated SOX17 and GATA-4 expression in keratinocytes (Fig. 6a). The activation of these endodermal markers was intriguing because in pancreas organogenesis in the embryo SOX 17 expression precedes that of PDX-1 [52]. The potential role of endodermal markers expression which is associated with the activation of the pancreatic lineage in PDX-1 treated keratinocytes should be further analyzed in future studies. On the other hand, SOX 17 expression may prevent further dedifferentiation into pluripotency as suggested by Niakan et al [53]. Despite its capacity to activate early endodermal markers, PDX-1 specifically activated endocrine pancreatic markers and not markers of other adult endodermal tissues, such as liver and exocrine pancreas (Fig. 6b).

The advantage of the transdifferentiation process described here is that it is relatively fast and is neither mediated by integration of foreign genetic information nor episodes of uncontrolled cell proliferation. Moreover, the generated cells are equipped by the desired characteristics and function, to be potentially used in cell therapy approaches. However, the total number of reprogrammed cells is restricted since most of the generated cells are post mitotic. To overcome the limited number of committed cells generated, the reprogramming efficiency should be further increased and the transdifferentiation process should be initiated in an abundant source of proliferating cells, which can be readily propagated prior to reprogramming induction.

In summary, transdifferentiation of ectoderm derived keratinocytes along the endodermal pancreatic lineage and function serves as a proof of concept to suggest that the transcription factors induced reprogramming is wider and more general developmental process than initially considered. The present study has enlarged the arsenal of potential cell sources suitable for regenerative medicine approaches, and future studies may utilize the most accessible and efficiently proliferating cells to be used as the desired source of tissue for cell therapy protocols.

## Supporting Information

Figure S1 **Lineage tracing for KRT5 positive cells (keratinocytes).** HaCaT cells (human keratinocytes cell line) were co-infected with the reporter vector (R/G) and either the constitutively active CMV-CRE vector (a–e, control) or the KRT5-CRE vector (f–j; keratinocyte-specific promoter activity). Seventy two hours (72 h) post infection, the cells express DsRed2 and/or eGFP. (a, f) Schematic presentation of the R/G reporter and Cre lentiviral vectors; (b, g) Phase-contrast images; (c, h) DsRed2 protein expression shows that many cells infected by the DsRed2/eGFP reporter alone or infected by both lentiviruses but, could not activate Cre; (d, i) eGFP protein expression shows the cells that were able to activate Cre. (e, j) Superimposed images show merged colors (yellow) where proteins are co-localized. Co-labeling with eGFP and DsRed2 likely reflects the activity of the Cre recombinase and the relatively long half-life of the DsRed2 protein (t_1/2_ = 4.5 days), such that the DsRed2 protein can be detected even 1–2 weeks after the DsRed2 gene is no longer expressed [34]. Original magnification ×20.
(TIF)

Methods S1 **Quantitative real-time RT-PCR primers list.** The primer sets used in this study are listed. Annealing temperate of all primers is 60°c.
(DOC)
