# Supplementary figures and images for: Ectopic PDX-1 Expression Directly Reprograms Human Keratinocytes along Pancreatic Insulin-Producing Cells Fate
Source: PLoS One. 2011 Oct 18;6(10):e26298. doi: 10.1371/journal.pone.0026298 (PMC3196540; doi:10.1371/journal.pone.0026298)

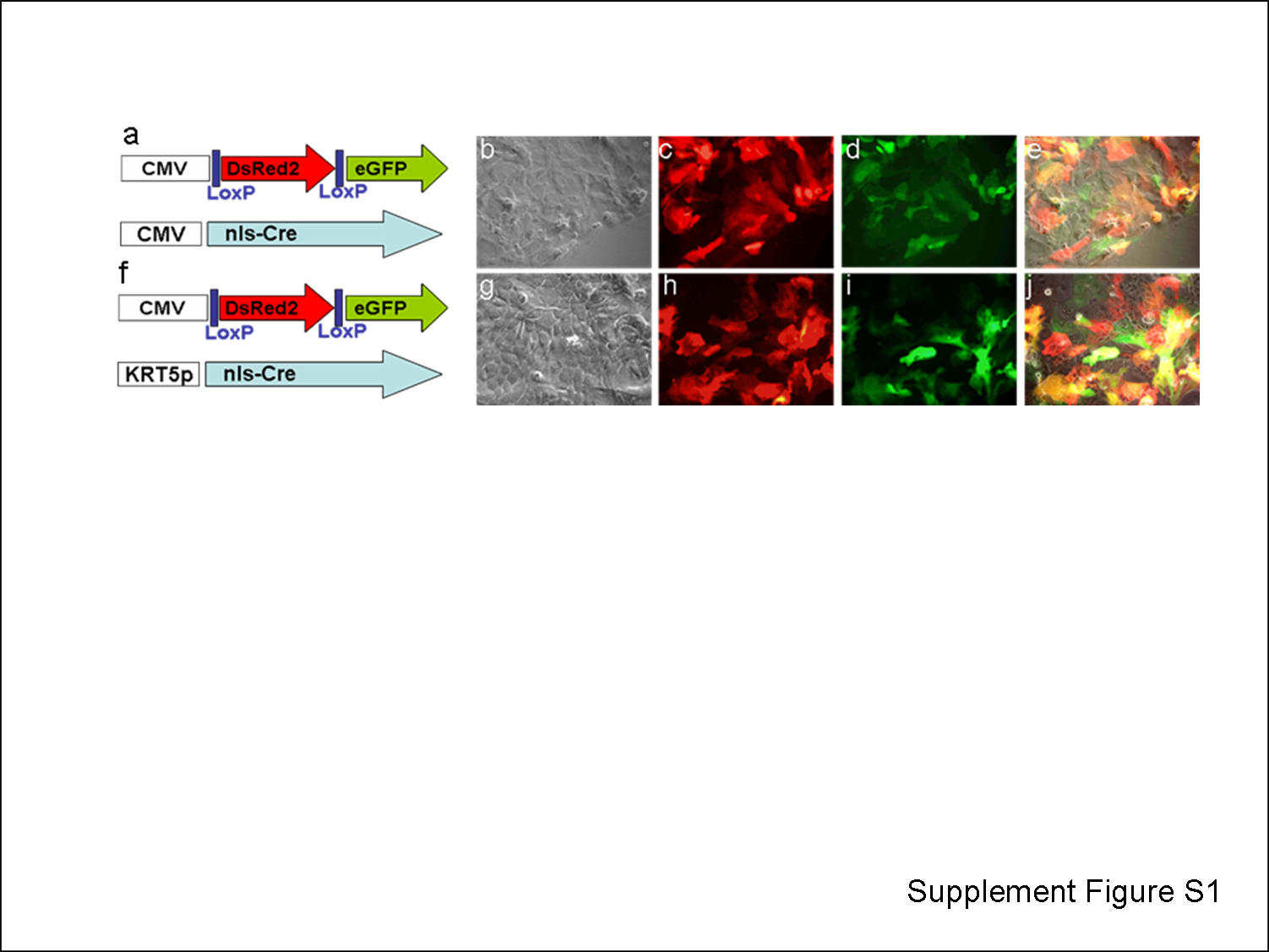

Supplement: Figure S1 — Lineage tracing for KRT5 positive cells (keratinocytes). HaCaT cells (human keratinocytes cell line) were co-infected with the reporter vector (R/G) and either the constitutively active CMV-CRE vector (a–e, control) or the KRT5-CRE vector (f–j; keratinocyte-specific promoter activity). Seventy two hours (72 h) post infection, the cells express DsRed2 and/or eGFP. (a, f) Schematic presentation of the R/G reporter and Cre lentiviral vectors; (b, g) Phase-contrast images; (c, h) DsRed2 protein expression shows that many cells infected by the DsRed2/eGFP reporter alone or infected by both lentiviruses but, could not activate Cre; (d, i) eGFP protein expression shows the cells that were able to activate Cre. (e, j) Superimposed images show merged colors (yellow) where proteins are co-localized. Co-labeling with eGFP and DsRed2 likely reflects the activity of the Cre recombinase and the relatively long half-life of the DsRed2 protein (t1/2 = 4.5 days), such that the DsRed2 protein can be detected even 1–2 weeks after the DsRed2 gene is no longer expressed [34]. Original magnification ×20. (TIF) [file pone.0026298.s001.tif]
