# Supplementary material for: Ectopic PDX-1 Expression Directly Reprograms Human Keratinocytes along Pancreatic Insulin-Producing Cells Fate
Source: PLoS One. 2011 Oct 18;6(10):e26298. doi: 10.1371/journal.pone.0026298 (PMC3196540; doi:10.1371/journal.pone.0026298)
Supplement: Methods S1 — Quantitative real-time RT-PCR primers list. The primer sets used in this study are listed. Annealing temperate of all primers is 60°c. (DOC) [file pone.0026298.s002.doc]

|  | F PRIMER | R PRIMER |
| --- | --- | --- |
| rat-PDX-1 | CCAGTT TGCAGGCTCGCTGG | GCTGCGTATGCACCTCCTGC |
| CGC | CCAAGATTTTGTGCAGTGGT | GGTAAAGGTCCCTTCAGCAT |
| GK | CATCTCTGAGTGCATCTCCGACT | TCGCAGTGATGGTCTTCGTAGTA |
| GLUT-2 | TCCAGCTACCGACAGCCTATT | CCAGCCGTCTGAAA AATGCT |
| INS | GCAGCCTTTGTGAACCAACA | CGGGTCTTGGGTGTGTAGAAGAAG |
| ISL1 | CGGGAGCCCTAATCCTCTCCCG | GCGGCGCAGCTGTTCTGATTA |
| MAF-A | AGCAGCGGCACAT TCTGG | TTGTACAGGTCC CGCTCTTTG |
| NEUROD-1 | ATGACCAAATCGTACAGCGAG | GTTCATGGCTTCGAGGTCGT |
| NGN-3 | ACCCCATTCTCTCTTCTTTTCTC CT | GAGGCGTCATCCTTTCTACCG |
| NKX2.2 | GGC CTT CAGTAC TCC CT | GGG ACT TGG AGC TTG AGT CCT |
| NKX6.1 | CGTTGGGGATGACAGAGAGT | CGAGTCCTGCTTCTTCTTGG |
| PAX-4 | CAGAGGCACTGGAGAAAGAGTTC | GGGCTTGAGACAGGCTTTAGG |
| PC1/3 | CTCTGGCTGCTGGCATCT | CTGCATATCTCGCCAGGTG |
| PC2 | GAGAAGACGCAGCCTACACC | CTGCAAAGCCATCTTTACCC |
| PDX-1 | CCATGGATGAAGTCTACCA | GTGCGCGTCCGCTTGTTCTC |
| SGNE-1 | GACCGGGTCTCAGAAGCAGATA | AGTCAACTCTGCCACGATGTT |
| SST | ATGATGCCCTGGAACCTGAAG | GCCGGGTTTGAGTTAGCAGAT |
| Tap63 | TTCTGAACAGCCTATATGTTCAG | TCTTCTGATGGTTCATCCACAA |
| ΔNp63 | TTTCCCACCCCGAGATGA | TGCGGCGAGCATCCAT |

Supplementary methods S1:
